# An AIE‐active bacterial inhibitor and photosensitizer for selective imaging, killing, and photodynamic inactivation of bacteria over mammalian cells

**DOI:** 10.1002/btm2.10539

**Published:** 2023-05-11

**Authors:** Fei Wang, Yupeng Shi, Po‐Yu Ho, Engui Zhao, Chuen Kam, Qiang Zhang, Xin Zhao, Yue Pan, Sijie Chen

**Affiliations:** ^1^ School of Science Harbin Institute of Technology, Shenzhen, HIT Campus of University Town Shenzhen China; ^2^ Ming Wai Lau Centre for Reparative Medicine Karolinska Institutet Hong Kong China; ^3^ Department of MRI The First Affiliated Hospital of Zhengzhou University Zhengzhou China; ^4^ Department of Biomedical Engineering The Hong Kong Polytechnic University Hong Kong China; ^5^ Guangdong Provincial Key Laboratory of Malignant Tumor Epigenetics and Gene Regulation, Guangdong‐Hong Kong Joint Laboratory for RNA Medicine, Medical Research Center, Sun Yat‐Sen Memorial Hospital Sun Yat‐Sen University Guangzhou China

**Keywords:** aggregation‐induced emission, bacterial inhibitor, photodynamic therapy, photosensitizer, wound healing

## Abstract

Photodynamic therapy is becoming increasingly popular for combat of bacteria. In the clinical photodynamic combat of bacteria, one critical issue is to avoid the potential damage to the host since the reactive oxygen species produced by photosensitizers are also harmful to mammalian cells. In this work, we report an aggregation‐induced‐emission‐active bacterial inhibitor and photosensitizer, OEO‐TPE‐MEM (OTM), for the imaging, killing, and light‐enhanced inactivation of bacteria. OTM could efficiently bind to and kill Gram‐positive bacteria, while its affinity to Gram‐negative bacteria is lower, and a higher OTM concentration is required for killing Gram‐negative bacteria. OTM is also an efficient photosensitizer and could efficiently sensitize the production of reactive oxygen species, which enhances its killing effect on both Gram‐positive and Gram‐negative bacteria. More interestingly, OTM is very biocompatible with normal mammalian cells both in the dark and under light irradiation. OTM in mice models with bacteria‐infected wounds could promote the healing of infected wounds without affecting their organs and blood parameters, which makes it an excellent candidate for clinical applications.

## 1 INTRODUCTION

Misuse of antimicrobial agents could potentially increase antibiotic resistance,^1^, ^2^, ^3^, ^4^, ^5^ which calls for novel methods for inactivation of bacteria. In recent years, antimicrobial photodynamic therapy (PDT) has been developed as an alternative therapy for microbial infection, especially skin and oral infections.^6^, ^7^, ^8^ A number of conventional fluorescent dyes, such as porphyrin and phenothiazine derivatives, are employed as photosensitizers (PSs) to transfer energy from light irradiation to oxygen for the generation of singlet oxygen (^1^O_2_) or other reactive oxygen species (ROS) in PDT.^9^ These toxic radicals can then induce cell death by apoptosis or necrosis. Despite of their wide usage in research and clinical practices, traditional fluorescent materials suffer from aggregation‐caused quenching effect which decreases both fluorescence and ROS‐sensitizing efficiency in the aggregated state and at high concentrations.^10^, ^11^ In contrast, materials with aggregation‐induced emission (AIE)^12^, ^13^, ^14^, ^15^, ^16^, ^17^ characteristics demonstrate both enhanced fluorescence and ROS‐sensitizing efficiency in the aggregated state and thus exhibit good performance in fluorescence imaging and great potentials in antimicrobial PDT.^18^, ^19^, ^20^, ^21^, ^22^ Increasing research effort has been devoted to exploring their potentials in imaging, biosensing, and PDT of tumors and microbes.^23^ Due to the feasibility of synthesis and excellent luminescence performance, tetraphenylethylene (TPE) and triphenylamine are widely used as AIE fluorophores to construct AIE‐active fluorescent materials.^24^

In the practical application of antibacterial PDT, one critical concern is the phototoxicity of PSs toward normal cells. Without cellular selectivity, PSs may also target mammalian cells and produce ROS upon light irradiation, which will destroy biomolecules in normal cells and lead to cell death. It is therefore important for the PSs to be highly selective toward bacteria or activatable in targets,^25^, ^26^, ^27^, ^28^, ^29^, ^30^ which could reduce the nonspecific toxicity. Increasing research effort has been devoted to minimizing nonspecific phototoxicity. To design a bacteria‐targeting AIE‐active PS, cationic groups are often linked to the AIE fluorophore core via π‐bridge(s) as a targeting group, which could attach to the negatively charged bacterial envelope by electrostatic interactions.^31^ The positively charged targeting groups also function as acceptors to construct a donor–acceptor system with the electron‐rich AIE core and π‐conjugation,^31^ and increase the hydrophilicity of the fluorogen as AIE cores are usually hydrophobic. Currently, monopositively charged pyridinium salt and dipositively charged pyridinium‐quaternary ammonium salts are widely used as targeting groups in bacterial membrane targeting AIE‐active probes.^32^, ^33^, ^34^, ^35^ Due to this amphipathic design, the AIE probes might also self‐assemble to form micelles or vesicles in aqueous conditions, which could influence the interaction of the probe with the bacterial envelope.^36^

**FIGURE 1:**
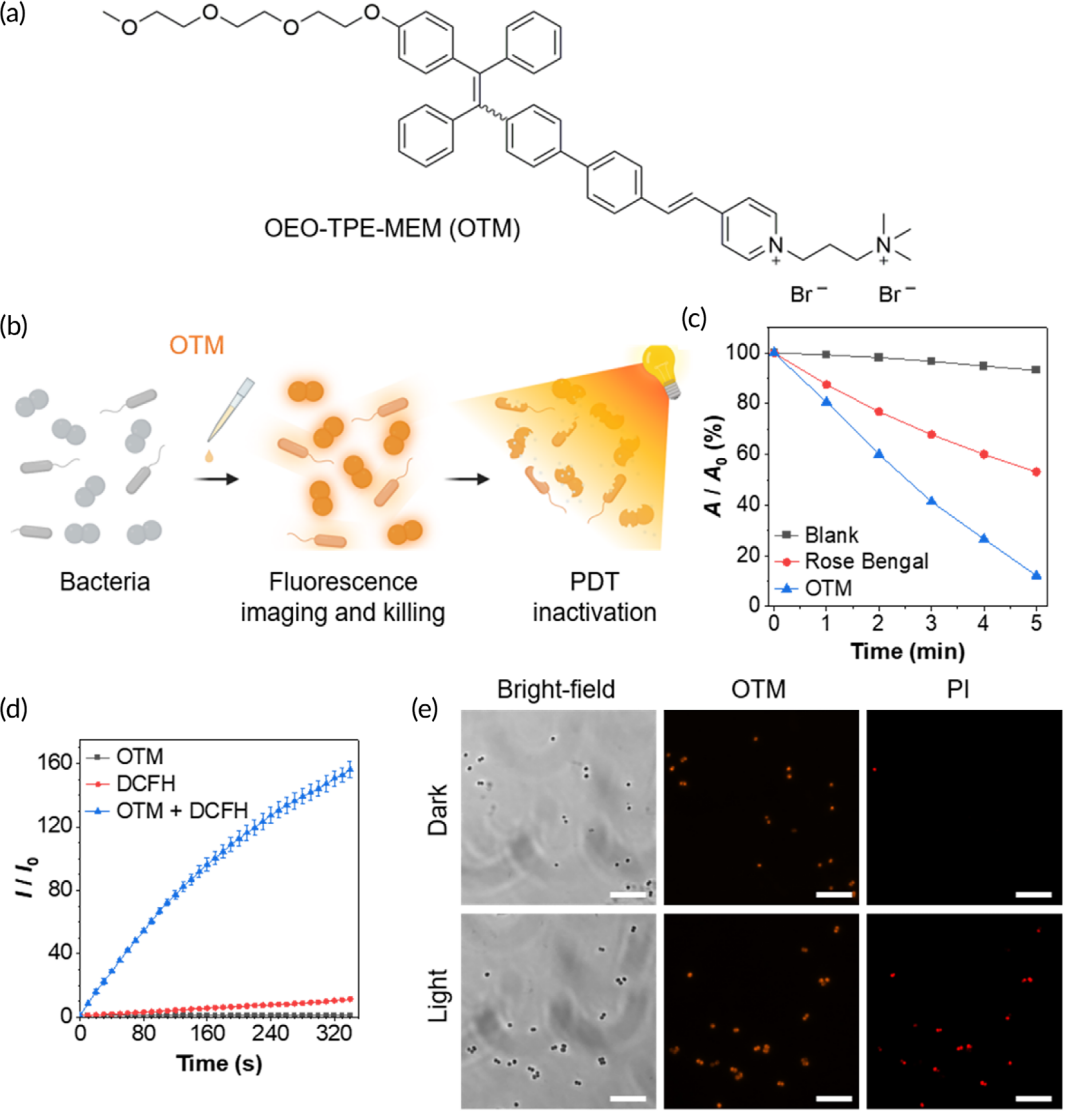
Photodynamic inactivation of Gram‐positive bacteria with OEO‐TPE‐MEM (OTM). (a) Molecular structure of OTM. (b) Schematic illustration of imaging, killing, and photodynamic inactivation of bacteria with OTM. (c) Detection of singlet oxygen (^1^O_2_) generated by light irradiation of OTM using ABDA. *A*: absorbance at 379 nm; *A*_0_: absorbance at 379 nm prior to light irradiation. (d) Detection of ROS generated by light irradiation of OTM using DCFH. *I*: fluorescence intensity at 525 nm; *I*_0_: fluorescence intensity at 525 nm prior to light irradiation. (e) Photodynamic killing of *Staphylococcus epidermidis* mediated by OTM. *S. epidermidis* was harvested and washed with pure water for three times, which was then stained with 5 μM OTM for 30 min in the dark. After staining, *S. epidermidis* was irradiated by white light (36 mW cm^−2^) for 10 min, followed by staining with PI. PI stained dead bacteria but not live bacteria. Scale bars: 10 μm. ABDA, 9,10‐anthracenediyl‐bis(methylene)dimalonic acid; DCFH, 2',7'‐dichlorofluorescin; PDT, photodynamic therapy; PI, propidium iodide.

Previously, we employed TPE‐based PSs for antibacterial PDT and achieved an excellent PDT effect on bacteria. Due to their amphiphilic nature, they stained both bacteria and mammalian cells. In this contribution, we rationally designed a new AIE‐active probe, OEO‐TPE‐MEM (OTM), by decorating the TPE core with a dipositively charged pyridinium‐quaternary ammonium salts and an amphipathic oligomeric ethylene glycol group (Figure 1a). Compared with the alkyl chain, the oligomeric ethylene glycol group possessed excellent solubility in both aqueous and organic solvents, which increased the water solubility of OTM and avoided its nonspecific interactions with mammalian cells. OTM demonstrated strong affinity toward Gram‐positive bacteria and acceptable affinity toward Gram‐negative bacteria, and its excellent water solubility enabled wash‐free staining of bacteria. OTM was an efficient bacterial inhibitor for both Gram‐positive and Gram‐negative bacteria. Besides, it could also efficiently sensitize the production of ROS upon light irradiation and could be applied to antibacterial PDT. Co‐staining of mammalian cells and bacteria revealed that OTM had strong selectivity to Gram‐positive bacterial membrane over Gram‐negative bacterial and mammalian cell membranes. This property endowed OTM with outstanding biocompatibility to be applied as a light‐enhanced bacterial inhibitor in vivo (Figure 1b).

## 2 MATERIALS AND METHODS

### 2.1 Materials and reagents

Dulbecco's Modified Eagle Medium (DMEM), fetal bovine serum (FBS), trypsin‐EDTA (0.25%), Dulbecco's phosphate buffered saline (DPBS), HBSS, LB broth, LB agar powder, and RB were purchased from Thermo Fisher Scientific (USA). NB, DCFH‐DA, and ABDA were purchased from Sigma‐Aldrich (USA). CCK‐8 was purchased from Yeasen (China). *E. coli* was purchased from China Center for Type Culture Collection (CCTCC). *S. epidermidis*, *S. aureus*, HeLa, HFF, and NIH/3T3 were purchased from American Type Culture Collection (ATCC). Pure water (18.2 MΩ cm) was obtained from a MilliQ system (Millipore, Germany). Glutaraldehyde was purchased from J&K Scientific (China).

### 2.2 Singlet oxygen detection

ABDA at a final concentration of 100 μM was used to monitor the generation of singlet oxygen (^1^O_2_). RB was used as the positive control, of which the quantum yield in pure water was documented as 0.75. A white‐light LED lamp was used for PDT at a power density of 36 mW cm^−2^. The absorption spectra of 300–420 nm was recorded with a SpectraMax M2e microplate reader (Molecular Devices) after irradiation for different periods of time to assess the elimination of ABDA.

### 2.3 ROS detection

Before each experiment, DCFH‐DA was freshly hydrolyzed by NaOH to generate DCFH. Briefly, 10 μL of 10 mM DCFH‐DA and 40 μL of 10 mM NaOH were mixed and incubated at room temperature for 30 min in the dark. Subsequently, 950 μL of PBS was added to neutralize and generate a stock solution of DCFH at 100 μM. A final concentration of 10 μM DCFH was used in ROS detection and a white‐light LED at a power density of 36 mW cm^−2^ was used for PDT. The resulted DCF was excited at 488 nm and the fluorescence intensity at 525 nm was detected with a SpectraMax M2e microplate reader (Molecular Devices) for measuring ROS production.

### 2.4 In vitro antimicrobial assays

OTM at final concentrations of 0.1–8 μM was added to 1 mL of bacteria with the final cell density at 1 × 10^8^ CFU/mL for *E. coli* and *S. epidermidis*, respectively, in pure water. Unstained bacteria were used as a control. All samples were incubated at room temperature for 30 min in the dark. Subsequently, a half of each sample was transferred to a 12‐well plate for light irradiation at a power density of 36 mW cm^−2^ for 10 min. The remaining half was the dark control. After light irradiation, 100 μL of each sample was diluted with a dilution factor of 100,000 and 100 μL of the diluted sample was spread on a LB agar plate and incubated in a 37°C incubator overnight. Images of the LB agar plates were captured by the camera on myECL Imager™ from Thermo Fisher Scientific (USA). To determine the growth curves of *S. epidermidis*, 4 × 10^7^ CFU/mL of *S. epidermidis* was inoculated into NB without (Control) or added with 10 μM OTM (OTM). Three replicates were prepared for each group. After the bacteria were cultured at 37°C with shaking at 250 rpm for desired time, 200 μL of each culture product were transferred to a well of a 96‐well plate to measure OD_600_ with a SpectraMax M2e microplate reader.

### 2.5 SEM observation

Bacteria were resuspended in 1 mL of pure water at the final bacterial cell density of 1 × 10^8^ CFU/mL for *E. coli* and 1 × 10^8^ CFU/mL for *S. epidermidis*, respectively. All samples were stained with 8 μM OTM at room temperature for 30 min in the dark. A half of each sample was transferred to a 12‐well plate for light irradiation at a power density of 36 mW cm^−2^ for 10 min. The remaining half was the dark control. All samples were collected by centrifugation at 8,000 ×*g* for 1 min at room temperature and washed twice with 66.7 mM phosphate buffer, pH 7.4. The samples were then fixed with 2.5% glutaraldehyde in 66.7 mM phosphate buffer, pH 7.4 at room temperature for 1 h following 4°C overnight. Samples were dehydrated with ethanol using a concentration gradient of 30–100%. After dehydration, samples were treated with ethanol/tert‐butanol mixture using a tert‐butanol concentration gradient of 30–100% (v/v), and were dropped on a silica slide, air‐dried, and sprayed with gold for SEM observation.

### 2.6 Cytotoxicity assays

NIH/3T3, HFF, and HeLa cells were used to test the cytotoxicity of OTM. All cells were cultured in DMEM supplemented with 10% FBS in a 37°C humidified incubator set to 5% CO_2_. Cells were trypsinized, resuspended in fresh complete medium, and seeded in 96‐well plates at a cell density of 12,000 cells per well for NIH/3T3 cells, 5,000 cells per well for HFF cells, and 7,000 cells per well for HeLa cells. Cells were cultured overnight. In the following day, the culture medium was replaced with fresh complete medium adding OTM at final concentrations of 2.5–20 μM. Complete medium without OTM was used as the untreated control. Cells were incubated in the incubator in darkness for 30 min. After incubation, cells were either irradiated with a white‐light LED lamp at a power density of 36 mW cm^−2^ for 10 min or kept in dark for 10 min. For staining–washing procedure, cells were then washed with PBS, changed to fresh complete medium and incubated in the incubator for another 24 h. For wash‐free procedure, cells were directly incubated in the incubator for another 24 h. After incubation, cells were changed to fresh complete medium with the CCK‐8 reagent and incubated for another 1 h in the incubator. The absorbance at 450 nm (A450) was measured by a SpectraMax M2e multimode microplate reader (Molecular Devices). Cell viability of each well was calculated as the percentage of A450 of the treated cells to A450 of the untreated control.

### 2.7 Wound healing assays

Animals received care following the guidance suggestions for the Care and Use of Laboratory Animals. The procedures were approved by the Animal Care and Use Committee (Ethics Committee of the Academy of Medical Sciences of Zhengzhou University) with a protocol code of ZZU‐LAC20220311.^14^ For each bacterial strain, 18 female BALB/c mice (6–8‐week‐old) were divided into three groups (PBS, OTM, and OTM + light) randomly with six for each group. On Day 0, one wound of 8 mm diameter was created by surgery scissors on the dorsal midline for each anesthetized mouse. Then, 20 μL of bacterial suspension (1 × 10^9^ CFU/mL) was inoculated on the surface of each wound for 12 h. On Day 1, each wound was treated with 20 μL of either PBS or 20 μM OTM solution in pure water for 30 min. After the treatment, the OTM + light group was irradiated with white light at a power density of ~7 mW cm^−2^ for 10 min. The treatment was performed once a day, repeatedly from Day 1 to Day 3. The sizes of each wound were measured with the aid of a ruler in the photographs. On Day 10, all wound and organ tissues were collected and subjected to staining, and the whole blood sample were also harvested. For each bacterial strain, whole blood samples of one mouse from PBS group, two mice from OTM and two mice from OTM + light group were tested by a BC‐2800VET Auto Hematology Analyzer from Mindray Animal Medical (China).

### 2.8 Hematoxylin and eosin staining

The granulation/callus and organ tissues fixed in 10% neutral buffered formalin were embedded in paraffin and 5‐mm‐thick tissue sections were obtained and stained with the standard H&E staining method. The stained sections were observed under a light microscope at desired magnification.

### 2.9 Statistical analysis

Quantitative analysis data are presented as mean ± standard deviation. All data are plotted with Origin lab software (USA).

## 3 RESULTS AND DISCUSSION

### 3.1 Applying OTM for antibacterial PDT

In our previous work, we systematically investigated the fluorescence imaging performance of OTM on Gram‐positive and Gram‐negative bacteria.^37^ The counter ion was changed from acetate to bromine in this study without influencing its selectivity as OTM was a cationic membrane probe. OTM can strongly bind to both Gram‐positive and Gram‐negative bacterial membranes in pure water. To explore the potential of employing OTM for antibacterial PDT, we first investigated the ^1^O_2_‐sensitizing ability of OTM in pure water by using 9,10‐anthracenediyl‐bis(methylene)dimalonic acid (ABDA) as an indicator. The absorbance of ABDA at 379 nm under light irradiation (36 mW cm^−2^) for 5 min was almost unchanged without PSs, while it was decreased by more than 90% in the presence of OTM (Figure 1c; Figure S1). For comparison, the photosensitizing ability of 5 μM of Rose Bengal (RB) was also investigated, in which the absorbance of ABDA at 379 nm was decreased by less than 50% under the same circumstances. These results indicated that OTM possessed higher ^1^O_2_ sensitizing efficiency than RB, which made OTM a promising PS candidate for antimicrobial PDT. Next, we examined the ROS‐sensitizing performance of OTM in PBS by using 2',7'‐dichlorofluorescin‐diacetate (DCFH‐DA) as a ROS indicator. As shown in Figure 1d, the fluorescence intensity at 525 nm after 350 s of white‐light irradiation was increased by less than 10‐fold with OTM or DCFH alone, while it was increased by over 140‐fold in the presence of both OTM and DCFH. These data suggested that OTM was very effective for sensitizing ROS production under light irradiation. Afterward, we examined the antibacterial PDT performance of OTM by staining it with propidium iodide (PI), which was a selective fluorescent probe for dead bacteria over live bacteria. Without light irradiation, almost all the bacteria were alive, while in the presence of light irradiation and OTM, most *S. epidermidis* were killed (Figure 1e). This result demonstrated that OTM was able to kill bacteria through PDT. The presence of OTM without light irradiation largely inhibited the growth of *E. coli* strain Nissle 1917 in MOPS minimal medium^37^ and completely inhibited the growth of *S. epidermidis* in Nutrient Broth (NB) (Figure S2). Besides, we also incubated *S. epidermidis* with OTM in the dark for 6 h and examined the viability of *S. epidermidis* by staining with PI (Figure S3). The *S. epidermidis* treated with OTM in the dark was stained by PI and showed red fluorescence. The earlier results suggested that OTM alone could kill *S. epidermidis* and function as a bacterial inhibitor, and white‐light irradiation could further enhance its antibacterial performance through PDT.

### 3.2 Light‐enhanced killing of both Gram‐positive and Gram‐negative bacteria with OTM

**FIGURE 2:**
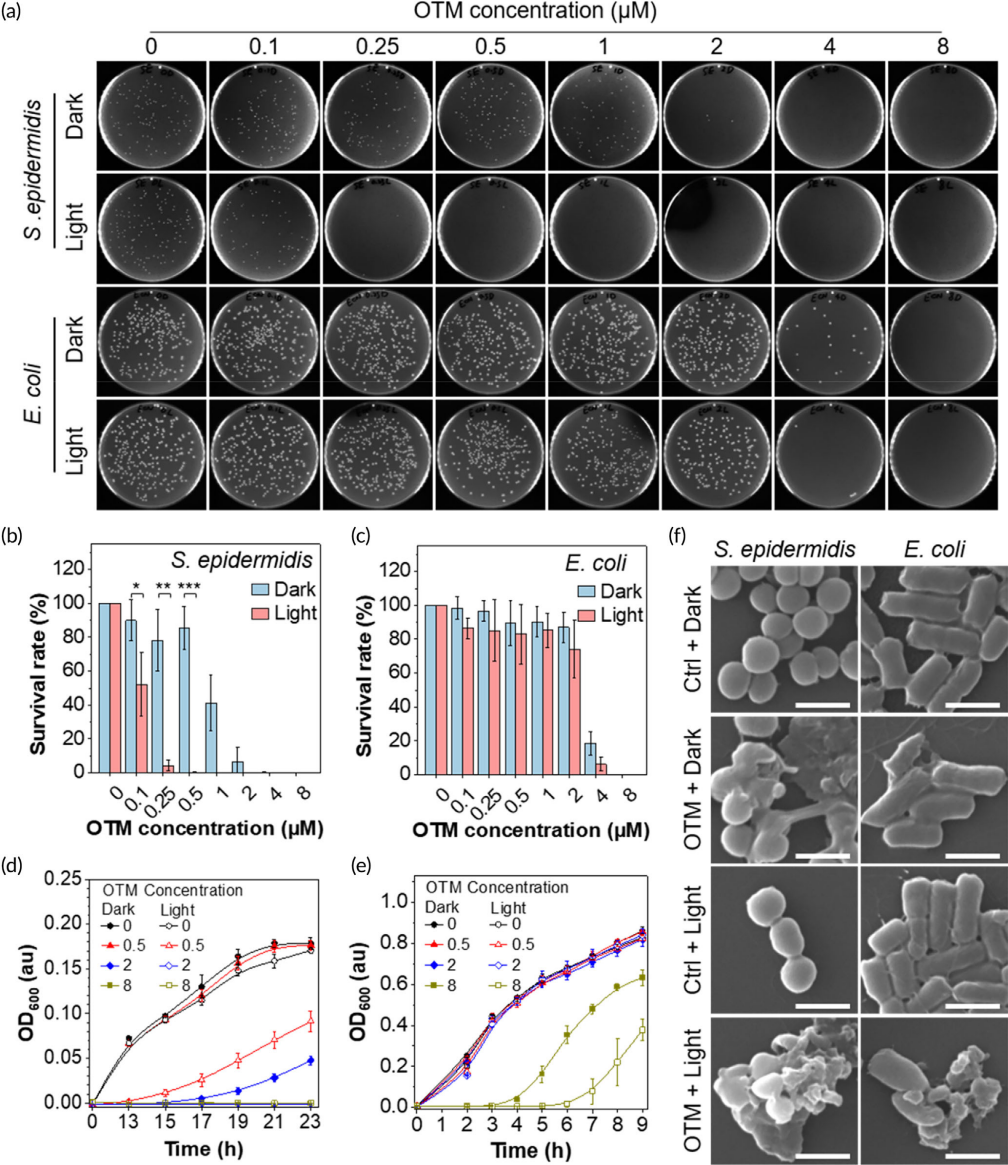
Determining the antibacterial effect of OEO‐TPE‐MEM (OTM) on both Gram‐positive and Gram‐negative bacteria in vitro. (a–c) Determination of the antibacterial effect of OTM by the plate count method. (a) Representative plates of the plate count assay. OTM of different concentrations (0, 0.1, 0.25, 0.5, 1, 2, 4, and 8 μM) were used to stain bacterial cultures for 30 min before spreading on the plates. (b and c) Statistical analysis of colony numbers of *Staphylococcus epidermidis* (b) and *Escherichia coli* (c) in the plate count assay. Three plates from three independent experiments were analyzed for each group, two‐tailed student's *t* test, *p < 0.05; **p < 0.01; ***p < 0.001. (d and e) Growth curves of OTM‐treated *S. epidermidis* in Nutrient Broth (NB) (d) and *E. coli* in Luria‐Bertani (LB) broth (e) with or without light irradiation. The concentration of OTM was indicated by the number in the figures. An equal volume of pure water was inoculated in the LB medium as a blank group. (f) Scanning electron microscope images of *S. epidermidis* and *E. coli* after OTM treatment with (Light) or without (Dark) light irradiation. Scale bars: 1 μm.

We next determined the PDT killing efficiency of *S. epidermidis* and *E. coli* by OTM using the plate count method. As shown in Figure 2a–c, bacteria could grow to form colonies at low‐OTM concentrations in the dark. As OTM concentration was increased, bacterial growth was gradually inhibited. The growth of *S. epidermidis* and *E. coli* were completely inhibited at OTM concentrations of 4 and 8 μM, respectively. White‐light irradiation greatly enhanced the bacterial killing effect of OTM on *S. epidermidis* with the lower inhibitory concentration of 0.25 μM (Figure 2a,b). Due to the weak affinity of OTM toward *E. coli*, white‐light irradiation only slightly increased its killing efficiency toward *E. coli* (Figure 2a,c). To validate the bacteria‐killing performance of OTM‐mediated antibacterial PDT, we determined the growth curve of OTM‐treated bacteria. As depicted in Figure 2d, OTM in the dark greatly extended the time of lag phase at concentrations of over 2 μM, indicating a high killing rate of *S. epidermidis*. Similar to the results in the plate count assay, white‐light irradiation lowered the inhibitory concentration of OTM to 0.5 μM (Figure 2d). As for *E. coli*, 8 μM of OTM extended the time of lag phase by 4 h, while white‐light irradiation could extend two more hours, which was corresponding to more than 60‐fold decrease in viable bacteria (Figure 2e). To directly observe the damage on bacteria caused by OTM, OTM‐treated bacteria were subjected to scanning electron microscope (SEM) analysis. As depicted in Figure 2f and Figure S4, OTM resulted in membrane deformation and fusion of bacterial envelopes in both *S. epidermidis* and *E. coli*. White‐light irradiation substantially enhanced the damage on bacteria envelopes. These results suggested that OTM could be used as a bacterial inhibitor against both Gram‐positive and Gram‐negative bacteria, and the killing efficiency could be further enhanced by PDT.

### 3.3 Selective staining of Gram‐positive bacteria under physiological conditions

**FIGURE 3:**
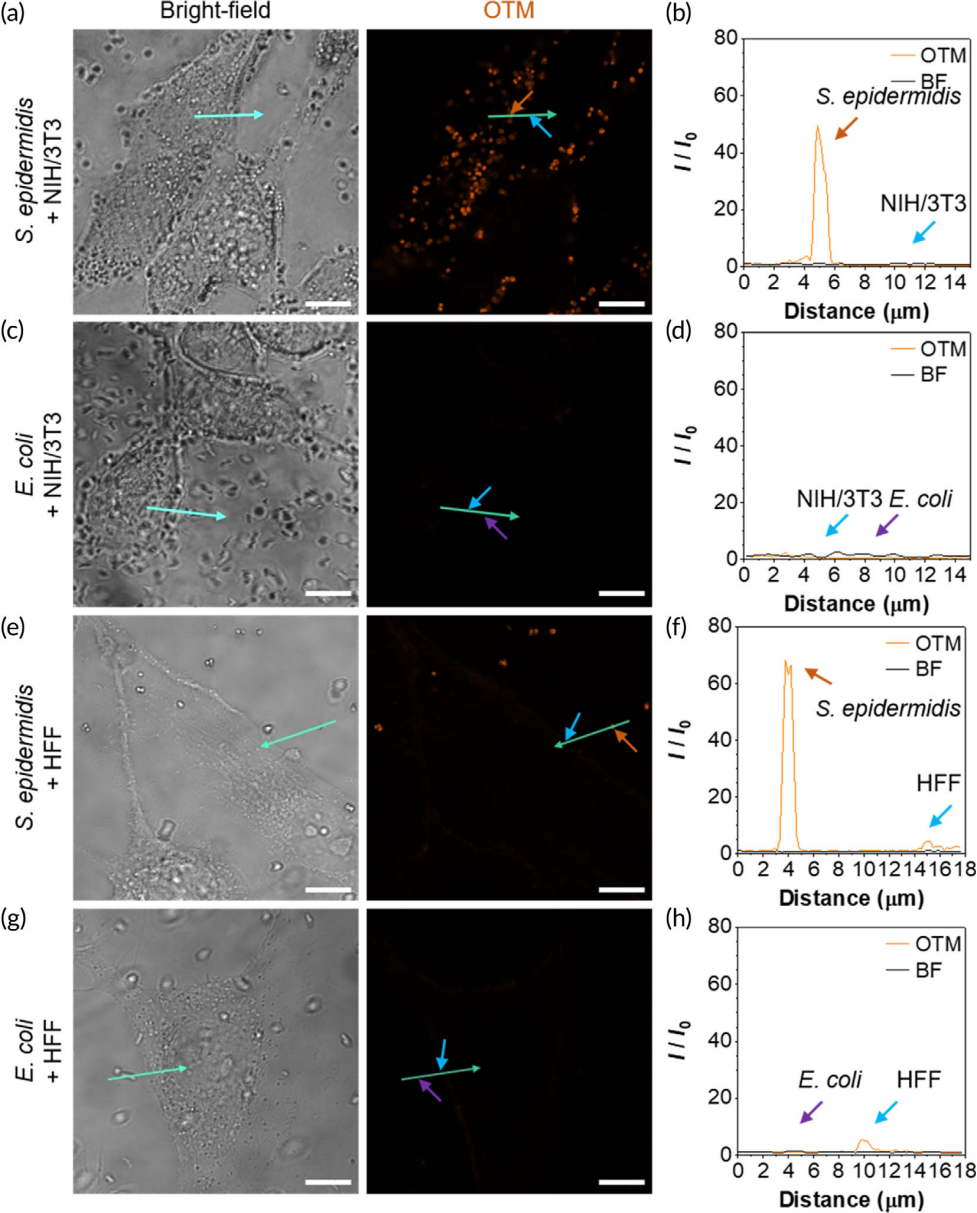
Selective staining of Gram‐positive bacteria over Gram‐negative bacteria and normal cells with OEO‐TPE‐MEM (OTM). (a, c, e, and g) Co‐staining of *Staphylococcus epidermidis* and NIH/3T3 cells (a), *Escherichia coli* and NIH/3T3 cells (c), *S. epidermidis* and HFF cells (e), *E. coli* and HFF cells (g) in HBSS buffer. Scale bars: 10 μm. (b, d, f, and h) Fluorescence intensities along the green arrows in panels (a, c, e, and g). Membranes of bacteria and mammalian cells were indicated by orange, purple, and blue arrows, respectively. BF, bright‐field. *I*_0_: fluorescence intensity at the starting point of the arrow. *I*: fluorescence intensity at given distance away from the arrow starting point.

To be an excellent candidate for antibacterial PDT applications, a PS should exert minimum interference on mammalian cells. We thus explored the selectivity of OTM. According to our previous work, the interaction of OTM with membranes could be affected by the ionic strength of solutions, which suggested its potential selectivity of bacteria over mammalian cells. To evaluate the affinity of OTM, we selected NIH/3T3 and HFF cell lines as models of normal mammalian cells. To mimic practical staining process under physiological conditions, cells and different bacteria were co‐stained with OTM in Hanks' balanced salt solution (HBSS) buffer with wash‐free procedures. After staining, the samples were imaged by confocal laser scanning microscope. As shown in Figure 3a,e, only *S. epidermidis* was strongly stained when NIH/3T3 or HFF cells were co‐stained with OTM. The fluorescence intensity of OTM was increased by more than 50‐fold upon interacting with the cell envelope of *S. epidermidis*, but it was not elevated or slightly elevated in the plasma membrane of NIH/3T3 or HFF cells (Figure 3b,f). Gram‐positive bacteria have the cell envelope of a thick outer peptidoglycan cell wall and an inner membrane of the phospholipid bilayer, whereas Gram‐negative bacteria have the cell envelope of a thin peptidoglycan cell wall sandwiched between an inner phospholipid bilayer and an asymmetric layer of lipopolysaccharides in the outer leaflet of the outer membrane.^38^, ^39^, ^40^, ^41^, ^42^ Outer membrane of Gram‐negative bacteria could therefore act as a barrier to impact the binding of compounds.^43^ When bound with cationic probes in PBS, ζ potential of the surface of Gram‐negative bacteria became higher than that of Gram‐positive bacteria,^44^, ^45^ which indicated the less affinity of cationic probes toward Gram‐negative bacteria in buffer saline. OTM showed much weaker affinity and less fluorescence sensitivity toward these Gram‐negative bacteria in HBSS buffer. When co‐stained with NIH/3T3 or HFF cells, OTM did not fluoresce upon interacting with the membrane of *E. coli* envelope or NIH/3T3 cells and weakly stained HFF cells (Figure 3c,d,g,h). To sum up, our data suggested that OTM selectively stained Gram‐positive bacteria over Gram‐negative bacteria and normal cells under physiological conditions, which facilitated its application as a biocompatible bacterial inhibitor in vivo.

### 3.4 Cytotoxicity of OTM

**FIGURE 4:**
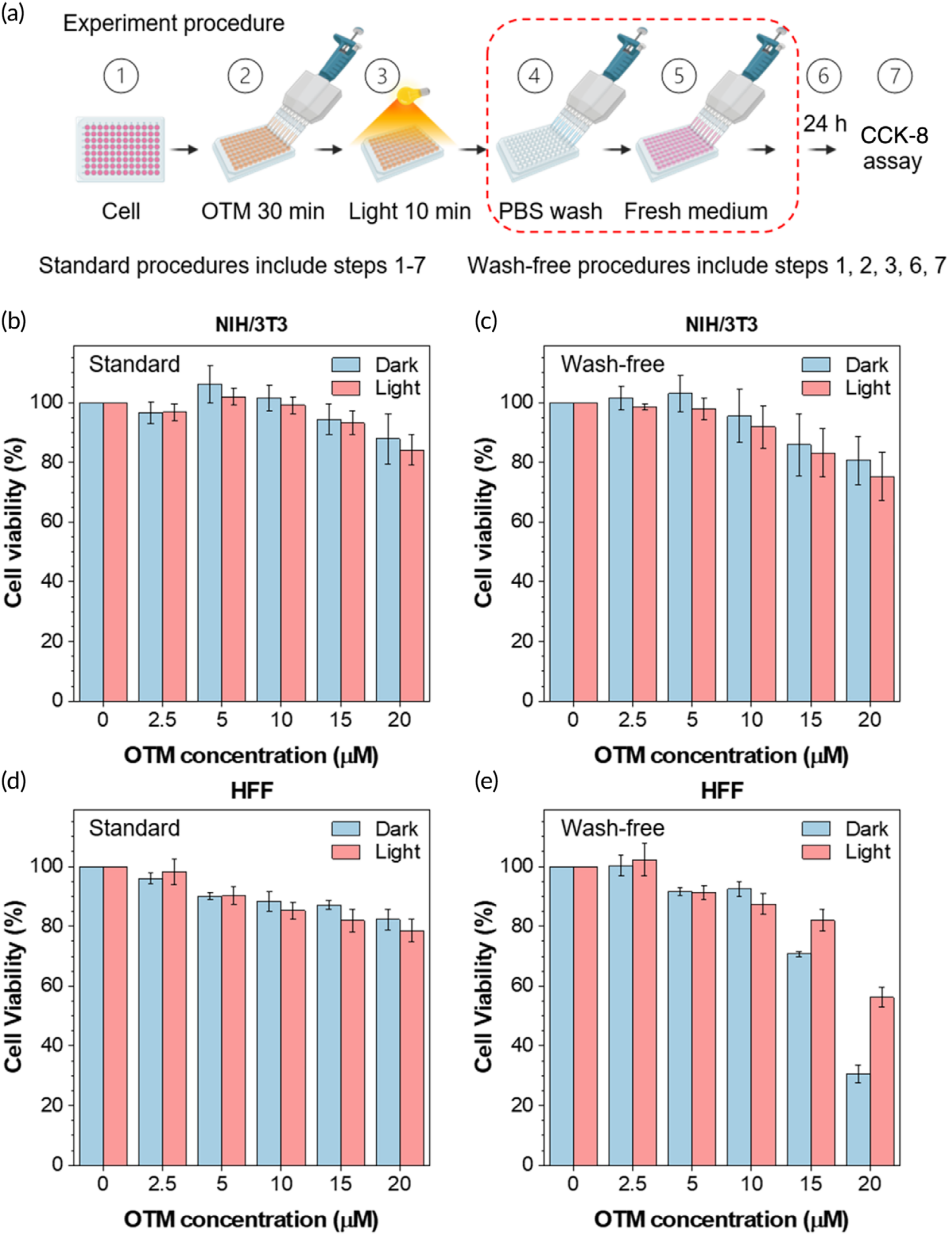
Determination of the cytotoxicity of OEO‐TPE‐MEM (OTM) on NIH/3T3 and HFF cells by the cell counting kit (CCK)‐8 assay. (a) Illustration of the standard procedures and wash‐free procedures for determination of OTM cytotoxicity. (b–e) Determination of the cytotoxicity of OTM with standard procedures (b and d) and wash‐free procedures (c and e) on NIH/3T3 or HFF cells by the CCK‐8 assay with (Light) or without light irradiation (Dark). Seven (0 μM) or eight replicates (2.5, 5, 10, 15, and 20 μM) in the same plate were measured for each group and three plates from three independent experiments were analyzed.

After determining the selectivity of OTM, we systematically measured the cytotoxicity of OTM on NIH/3T3 and HFF cells with or without light irradiation. In standard procedures, cells were incubated with different concentrations (0, 2.5, 5, 10, 15, and 20 μM) of OTM for 30 min in the incubator (Figure 4a). After incubation, the cells were irradiated by white light (36 mW cm^−2^) for 10 min at room temperature, while cells in control groups were kept in the dark for 10 min. Then, all cells were washed with PBS once to remove the unbound OTM in the culture medium and incubated in a fresh culture medium. After incubation for 24 h in the incubator, cell viability was measured by the CCK‐8 assay. As shown in Figure 4b, OTM exerted neglectable influence on NIH/3T3 cells with 84% cell viability at a concentration of 20 μM in the light‐irradiated group. Light irradiation did not affect cell viability of NIH/3T3 cells. We also investigated the influence of OTM on cell viability without washing away unbound molecules. As shown in Figure 4c, more than 75% of cells remained viable at 20 μM of OTM and in the presence of light irradiation. Similar to the results on NIH3T3 cells, OTM treatment with standard procedure had minimal influence on HFF cells with more than 78% cell viability at the concentration of 20 μM without or with light irradiation (Figure 4d). Although the wash‐free procedure slightly increased toxicity to HFF cells, more than 70% of cells remained viable at 15 μM of OTM in the presence of light irradiation (Figure 4e), which exceeded the concentration needed for antibacterial PDT. As cancer cells usually had higher affinity to cationic probes, we also tested the selectivity and biocompatibility in a cancer cell line (HeLa). Unlike normal cells, OTM showed much stronger affinity (Figure S5) and toxicity (Figure S6) toward cancer cells. Taken together, our results demonstrated the good biocompatibility of OTM toward normal cells, which was beneficial for in vivo application of antimicrobial PDT.

### 3.5 OTM for light‐enhanced antibacterial therapy against *E. coli* and *S. aureus* in vivo

**FIGURE 5:**
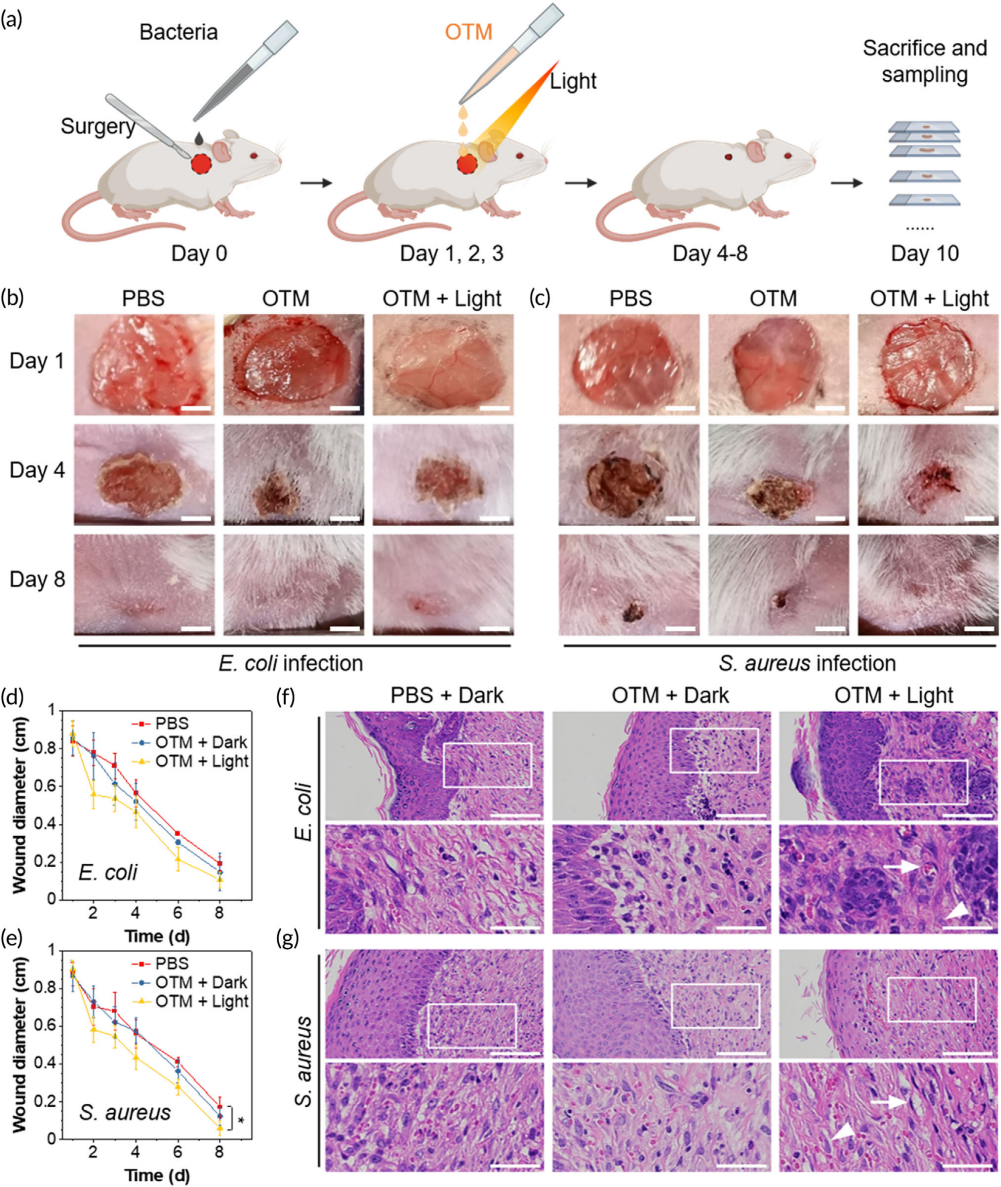
The wound healing assay in mice treated with OEO‐TPE‐MEM (OTM)‐mediated photodynamic therapy (PDT). (a) Schematic illustration of experimental procedures of the wound healing assay in mice to evaluate the effect of OTM‐mediated PDT. (b and c) Evaluation of the in vivo antimicrobial effect of OTM‐mediated PDT against *Escherichia coli* (b) and *Staphylococcus aureus* (c) infections in mouse wounds. Scale bars: 2 mm. (d and e) Statistic analysis of healing speed of bacteria‐infected mouse wounds in b (d) and c (e). One wound was created in each mouse and six mice were used in each group, two‐tailed Student's *t*‐test, *p < 0.05. (f,g) Hematoxylin and eosin (H&E) staining of *E. coli* infected (f) and *S. aureus* infected (g) wounds in the presence of PBS, OTM without light irradiation (OTM + Dark), or OTM with white‐light irradiation (OTM + Light). Blood vessels and fibroblasts are indicated by arrows and arrowheads, respectively. Scale bars in original images: 100 μm; scale bars in magnified images: 40 μm.

**FIGURE 6:**
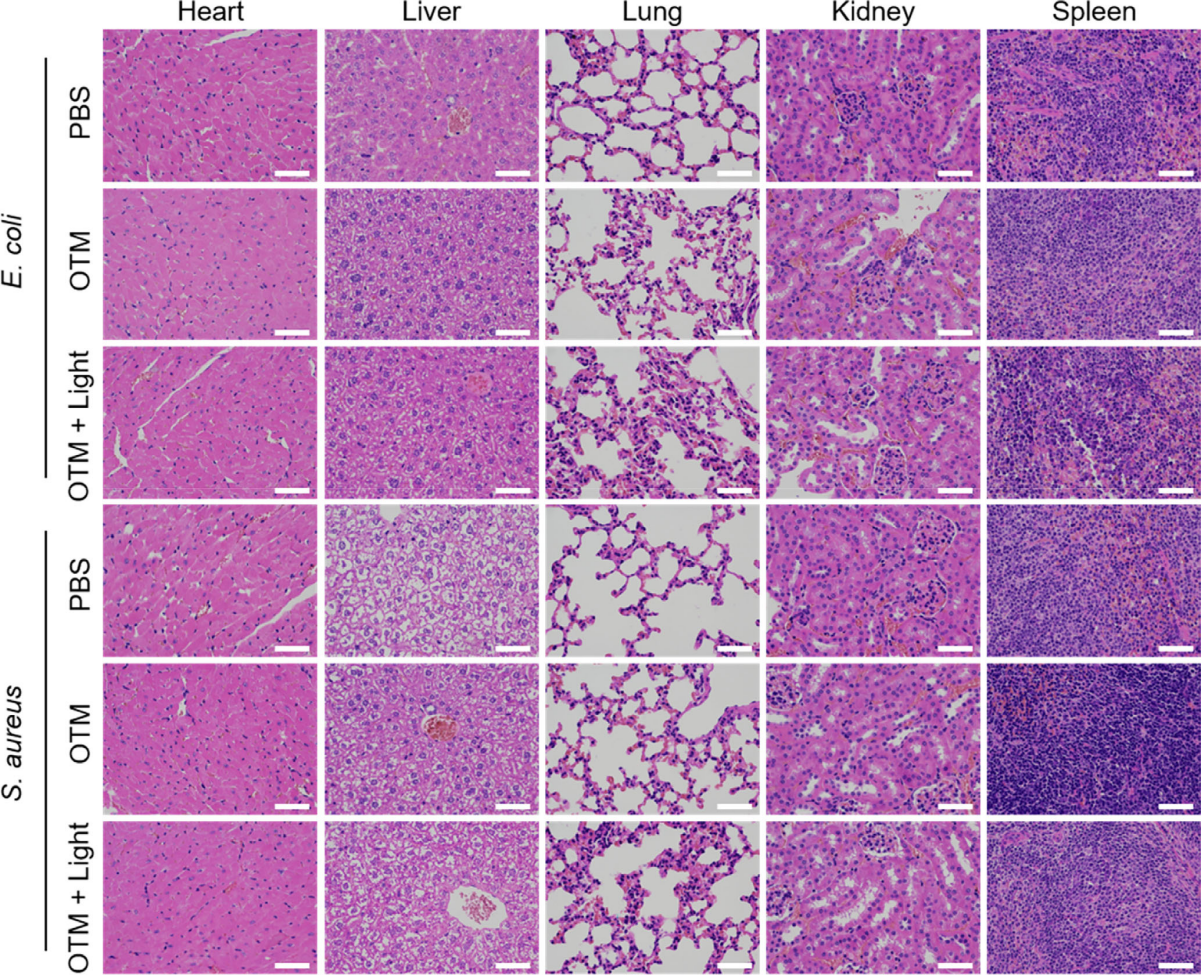
H&E staining of different tissues (heart, liver, lung, kidney, and spleen) from bacterial‐infected mice in the wound healing assay treated with or without OTM‐mediated PDT. Scale bars: 100 μm. OTM, OEO‐TPE‐MEM; PDT, Photodynamic therapy.

To explore the practical applications of OTM, wound healing assays were performed to evaluate light‐enhanced inhibition of bacteria in vivo by OTM in the mouse model with wounds inflicted on the dorsal back skin. Because *E. coli* strain Nissle 1917 was probiotic and *S. epidermidis* was a commensal bacterium of the skin, two other pathogenic strains, *E. coli* and *S. aureus*, were used as representatives of Gram‐negative and Gram‐positive bacteria in the wound healing assay. Wounds infected with *E. coli* or *S. aureus* were treated with OTM in the presence/absence of light irradiation. PBS treatment was employed as a control group. The experiment procedures are illustrated in Figure 5a. Briefly, one wound was surgically created on each mouse with inoculation of equal number of bacteria on Day 0. After infection, all groups showed a similar degree of abscess before treatment (Day 1). Then, the wounds were treated by PBS or OTM with the PDT method once a day from Day 1 to Day 3. As demonstrated in Figure 5b,c, wounds treated with OTM in PDT showed substantial decrease in wound diameters in Day 4 and Day 8 compared with the control group. Statistical analysis of bacterial‐infected wounds revealed that white‐light irradiation enhanced the antibacterial effect of OTM (Figure 5d,e). On Day 10, the hematoxylin and eosin (H&E) staining showed that fibroblasts and blood vessels were newly formed in PDT‐treated wounds (Figure 5f,g), indicating that PDT using OTM promoted the wound healing process. To investigate the potential damage caused by OTM, we harvested and observed the organs from the control and PDT‐treated mice with H&E staining. As shown in Figure 6, OTM treatment with or without light irradiation did not cause any additional damage in mouse heart, liver, lung, kidney, or spleen. Furthermore, the whole blood analysis confirmed that OTM‐mediated PDT did not cause any abnormality in mouse blood parameters (Table S1). Taken together, our results demonstrated the excellent biocompatibility and feasibility of using OTM in PDT for bacterial skin infections.

## 4 CONCLUSION

In summary, we developed an AIE‐active bacterial inhibitor (OTM) against both Gram‐positive and Gram‐negative bacteria, of which the antibacterial effect could be further enhanced by light irradiation. We systematically studied the antibacterial process of OTM. In the absence of light irradiation, OTM completely inhibited the formation of *S. epidermidis* or *E. coli* colonies at relatively low concentrations (4 and 8 μM, respectively), which enabled its application as an effective bacterial inhibitor. Besides, OTM demonstrated great abilities to sensitize ROS production under light irradiation, which greatly improved its antibacterial effect through PDT and lowered its inhibitory concentration under light irradiation. In the presence of light, 0.5 μM of OTM could completely inhibit the colony formation of *S. epidermidis*. More importantly, OTM showed excellent aqueous solubility and strong selectivity to Gram‐positive bacteria over mammalian cells, which enhanced the biosafety when used as a bacterial inhibitor in vivo with a simplified wash‐free procedure. The in vivo antibacterial experiment of bacteria‐infected wounds in mice showed that OTM‐mediated PDT accelerated wound healing in mice without any adverse effects. All these results demonstrated that OTM possessed both advantages of easy‐to‐use and extraordinary biosafety. The current work paves the way for the development and clinical application of AIE‐active PSs for antimicrobial PDT in the future.

## AUTHOR CONTRIBUTIONS

**Fei Wang:** Investigation (lead); methodology (equal); data curation (lead); writing – original draft (lead); writing – review and editing (lead). **Yupeng Shi:** Investigation (equal); methodology (equal); data curation (equal). **Po‐Yu Ho:** Writing – original draft (equal); methodology (equal). **Engui Zhao:** Funding acquisition (lead); conceptualization (lead); writing–original draft (lead); writing–review and editing (lead). **Chuen Kam:** Writing – original draft (equal); writing – review and editing (equal). **Qiang Zhang:** Methodology (equal). **Xin Zhao:** Methodology (equal). **Yue Pan:** Methodology (equal). **Sijie Chen:** Funding acquisition (lead); conceptualization (lead); supervision (lead); writing – review and editing (lead).

## CONFLICT OF INTEREST STATEMENT

The authors declare no competing financial interests.

## Supporting information


**Data S1:** Supporting information.

## Data Availability

All the data are present in the main article and Supplemental information.
