# Supplementary material for: An AIE‐active bacterial inhibitor and photosensitizer for selective imaging, killing, and photodynamic inactivation of bacteria over mammalian cells
Source: Bioeng Transl Med. 2023 May 11;8(6):e10539. doi: 10.1002/btm2.10539 (PMC10658525; doi:10.1002/btm2.10539)
Supplement: Supplementary file 1 — Data S1: Supporting information. [file BTM2-8-e10539-s001.docx]

*Supporting Information*

An AIE-active bacterial inhibitor and photosensitizer for selective imaging, killing and photodynamic inactivation of bacteria over mammalian cells

Fei Wang^1,2,‖^, Yupeng Shi^2,3,‖^, Po-Yu Ho^2^, Engui Zhao^1^, Chuen Kam^2^, Qiang Zhang^4^, Xin Zhao^4^, Yue Pan^5^, Sijie Chen^2^

^1^School of Science, Harbin Institute of Technology, Shenzhen, HIT Campus of University Town, Shenzhen 518055, P. R. China

^2^Ming Wai Lau Centre for Reparative Medicine, Karolinska Institutet, Hong Kong 999077, P. R. China

^3^Department of MRI, The First Affiliated Hospital of Zhengzhou University, Zhengzhou 450052, P. R. China

^4^Department of Biomedical Engineering, The Hong Kong Polytechnic University, Hong Kong 999077, P. R. China

^5^Guangdong Provincial Key Laboratory of Malignant Tumor Epigenetics and Gene Regulation, Guangdong-Hong Kong Joint Laboratory for RNA Medicine, Medical Research Center, Sun Yat-Sen Memorial Hospital, Sun Yat-Sen University, Guangzhou 510120, P. R. China

^‖^Fei Wang and Yupeng Shi contributed equally to the work.

**Correspondence**

Engui Zhao, School of Science, Harbin Institute of Technology, Shenzhen, HIT Campus of University Town, Shenzhen 518055 P. R. China

E-mail: zhaoengui@hit.edu.cn

Sijie Chen, Ming Wai Lau Centre for Reparative Medicine, Karolinska Institutet, Hong Kong 999077, P. R. China

E-mail: sijie.chen@ki.se


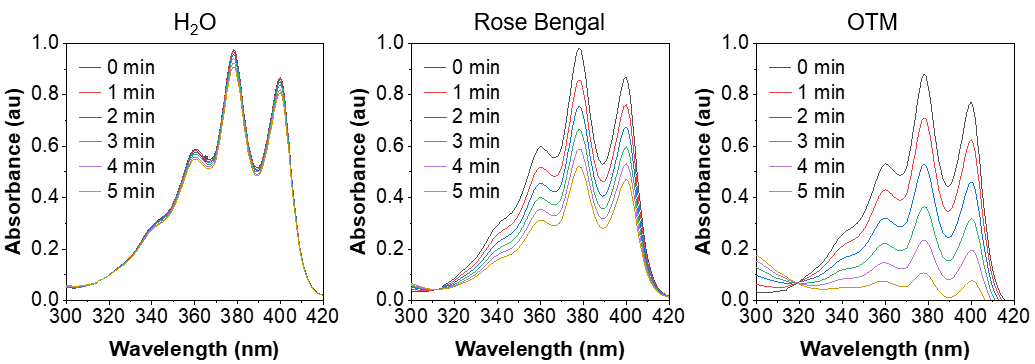


**Figure S1**. Detection of singlet oxygen generation of RB and OTM under light irradiation in pure water using ABDA as indicator.


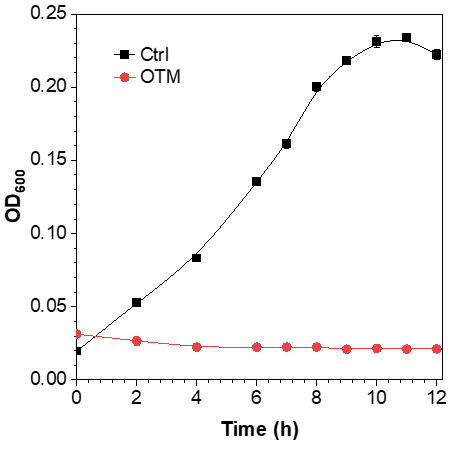


**Figure S2.** Growth curves of *S. epidermidis* in NB in the absence (Ctrl) or presence (OTM) of 10 µM OTM.


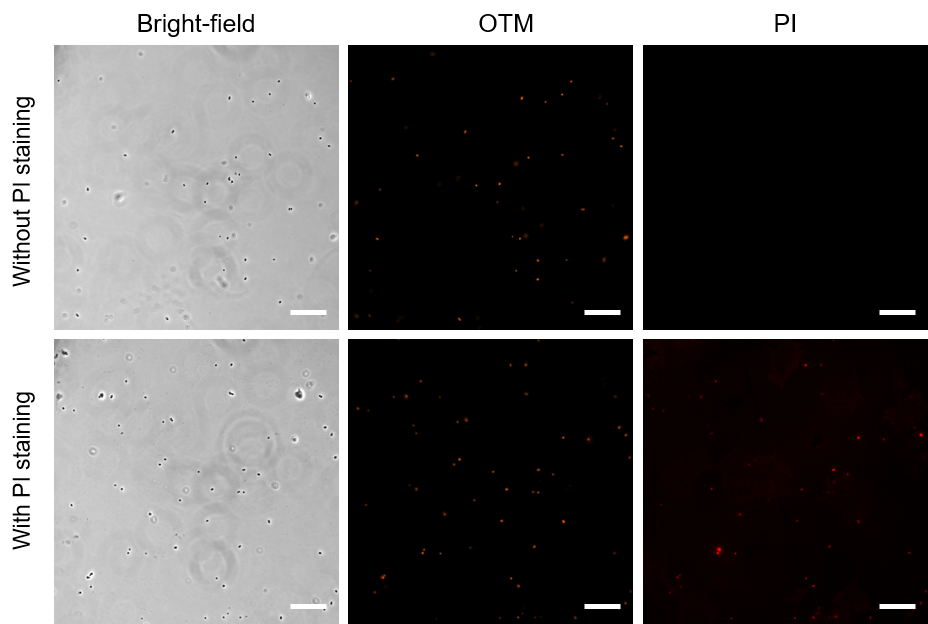


**Figure S3.** Killing of *S. epidermidis* by 6-h OTM staining. Dead bacteria were stained by PI, while living bacteria were not. Scale bars: 10 µm.

**
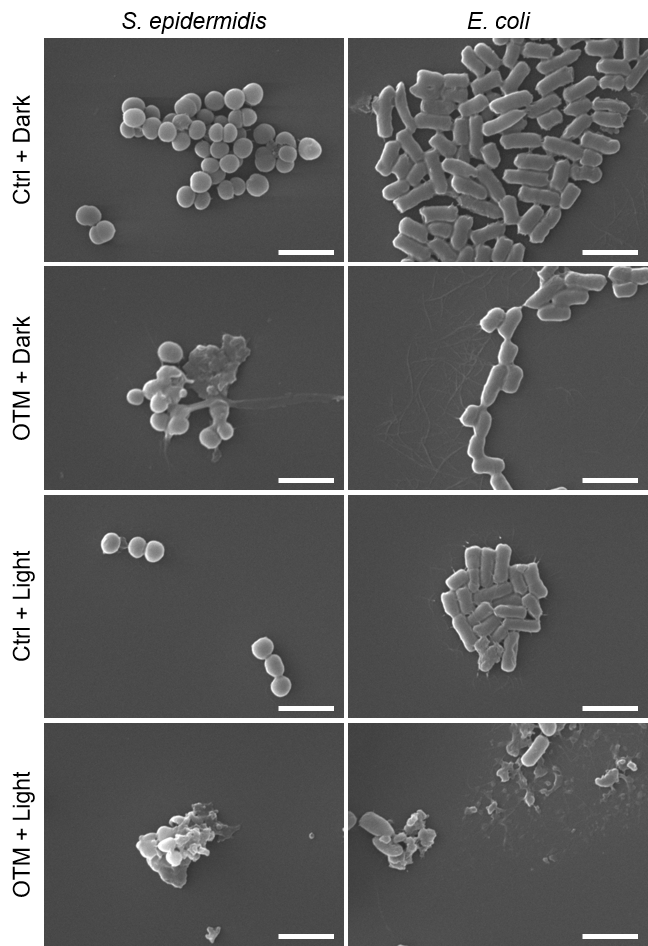
**

**Figure S4**. TEM pictures in Figure 2F with lower magnifications. Scale bars: 2 μm.


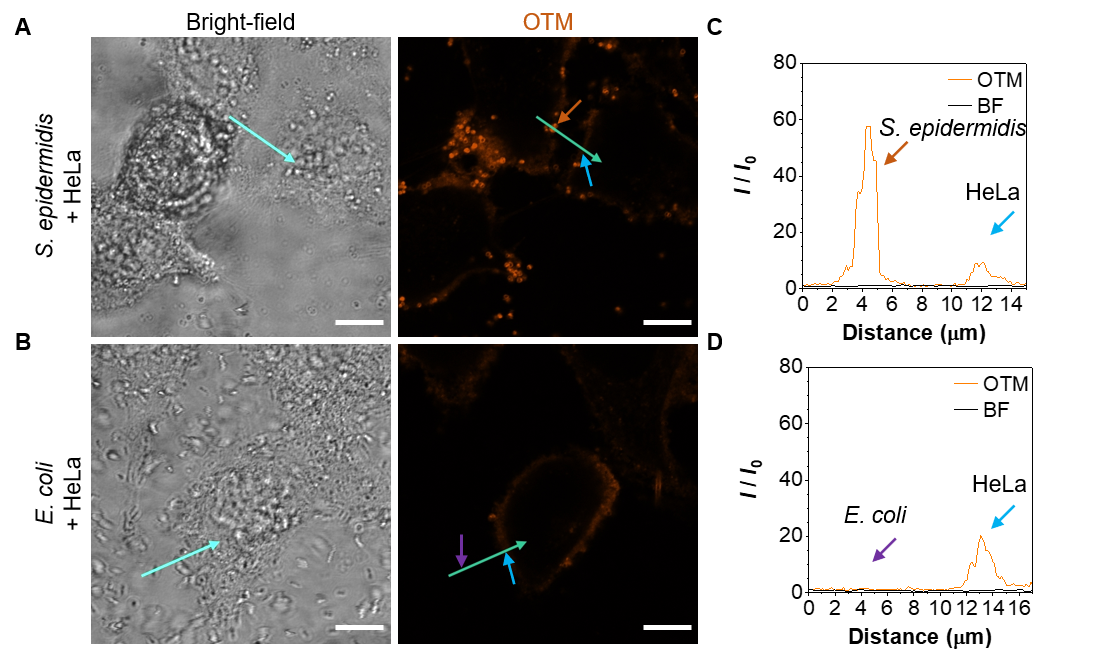


**Figure S5**. Staining of Gram-positive bacteria and HeLa cells over Gram-negative bacteria with OTM.

(A and C) Co-staining of *S. epidermidis* and HeLa cells (A) and *E. coli* and HeLa cells (C) in HBSS buffer. Scale bars: 10 µm. (B and D) Fluorescence intensities along the green arrows in panels (A) and (C). Membranes of bacteria and HeLa cells were indicated by orange, purple and blue arrows, respectively. BF, bright-field. *I*_0_: fluorescence intensity at the starting point of the arrow. *I*: fluorescence intensity at given distance away from the arrow starting point.


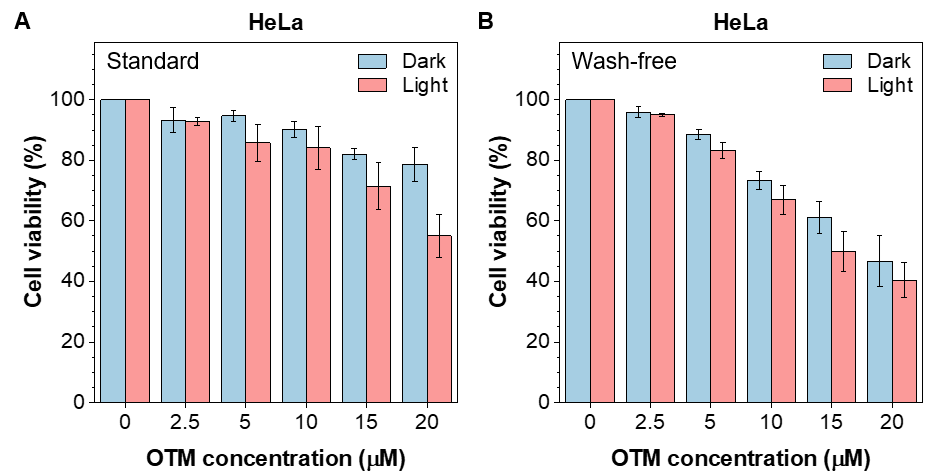


**Figure S6**. Determination of the cytotoxicity of OTM on HeLa cell by the CCK-8 assay.

**Table S1**. Whole blood analysis of blood samples from *E. coli* and *S. aureus* infected mice, treated with or with OTM mediated PDT.

| *Group Parameters* | *E. coli* | | | | |  | *S. aureus* | | | | | Unit | Normal values |
| --- | --- | --- | --- | --- | --- | --- | --- | --- | --- | --- | --- | --- | --- |
|  | PBS | OTM  Dark #1 | OTM  Dark #2 | OTM  Light #1 | OTM  Light #2 |  | PBS | OTM  Dark #1 | OTM  Dark #2 | OTM  Light #1 | OTM  Light #1 |  |  |
| WBC | 3 | 1.7 | 3.7 | 3.7 | 1.7 |  | 2 | 2.5 | 1.1 | 2.3 | 4 | 10^9/L | 0.8-6.8 |
| Lymph# | 2.3 | 1.2 | 2.9 | 2.5 | 1.3 |  | 1.8 | 1.9 | 0.5 | 0.9 | 3.2 | 10^9/L | 0.7-5.7 |
| Mon# | 0.1 | 0.1 | 0.1 | 0.2 | 0 |  | 0 | 0.1 | 0.2 | 0.3 | 0.1 | 10^9/L | 0.0-0.3 |
| Gran# | 0.6 | 0.4 | 0.7 | 1 | 0.4 |  | 0.2 | 0.5 | 0.4 | 1.1 | 0.7 | 10^9/L | 0.1-1.8 |
| Lymph% | 76.9 | 70.4 | 78.3 | 67.4 | 74.2 |  | 90.1 | 76.3 | 47.8 | 41.3 | 79 | % | 55.8-90.6 |
| Mon% | 3.8 | 5.3 | 3.5 | 6.4 | 5.1 |  | 1.9 | 5 | 14.7 | 12.6 | 2.9 | % | 1.8-6.0 |
| Gran% | 19.3 | 24.3 | 18.2 | 26.2 | 20.7 |  | 8 | 18.7 | 37.5 | 46.1 | 18.1 | % | 8.6-38.9 |
| RBC | 9.41 | 6.84 | 10.2 | 5.25 | 6.68 |  | 5.33 | 9.48 | 3.5 | 6.97 | 9.9 | 10^12/L | 6.36-9.42 |
| HGB | 142 | 109 | 157 | 79 | 105 |  | 81 | 147 | 52 | 115 | 147 | g/L | 110-143 |
| HCT | 44 | 32.1 | 47.7 | 25.2 | 30.9 |  | 26.3 | 44.4 | 16.7 | 32.8 | 45.7 | % | 34.6-44.6 |
| MCV | 46.8 | 47 | 46.8 | 48.1 | 46.4 |  | 49.5 | 46.9 | 47.8 | 47.1 | 46.2 | fL | 48.2-58.3 |
| MCH | 15 | 15.9 | 15.3 | 15 | 15.7 |  | 15.1 | 15.5 | 14.8 | 16.4 | 14.8 | Pg | 15.8-19 |
| MCHC | 322 | 339 | 329 | 313 | 339 |  | 307 | 331 | 311 | 350 | 321 | g/L | 302-353 |
| RDW | 15.9 | 17.6 | 17 | 20.8 | 17 |  | 20.4 | 17 | 19.2 | 18 | 14.9 | % | 13-17 |
| PLT | 604 | 142 | 414 | 294 | 83 |  | 136 | 82 | 82 | 69 | 445 | 10^9/L | 450-1590 |
| MPV | 5.6 | 6.5 | 6.5 | 6 | 5.6 |  | 5.7 | 6.5 | 6 | 6.2 | 6 | fL | 3.8-6.0 |
| PDW | 16.8 | 17.8 | 17.4 | 17.8 | 17.6 |  | 18.9 | 17.5 | 19.3 | 18.2 | 16.9 |  |  |
| PCT | 0.338 | 0.092 | 0.269 | 0.176 | 0.046 |  | 0.077 | 0.053 | 0.049 | 0.042 | 0.267 | % |  |
